# Vision-related quality of life in patients receiving intravitreal ranibizumab injections in routine clinical practice: baseline data from the German OCEAN study

**DOI:** 10.1186/s12955-016-0536-1

**Published:** 2016-09-20

**Authors:** Thomas Bertelmann, Nicolas Feltgen, Martin Scheffler, Ulrich Hufenbach, Annette Wiedon, Helmut Wilhelm, Focke Ziemssen

**Affiliations:** 1 Department of Ophthalmology, University Medical Center Goettingen, Robert-Koch-Straße 40, 37075 Goettingen, Germany; 2 MCR-Medical Center Rhauderfehn, Rhauderfehn, Germany; 3 Wernigerode, Germany; 4 Novartis Pharma GmbH, Nuremberg, Germany; 5 Centre for Ophthalmology, Eberhard-Karl University, Tuebingen, Germany

**Keywords:** Age-related macular degeneration, Diabetic macular edema, Retinal vein occlusion, Real-world evidence, Ranibizumab, OCEAN study, Anti-VEGF, Intravitreal injection, Visual acuity (VA), Optical coherence tomography (OCT)

## Abstract

**Background:**

Vision-related quality of life (vrQoL) is advancing more and more into the focus of interest in ophthalmological clinical research. However, to date only little information is available about vrQoL from large non-interventional studies in terms of "real-world evidence". The purpose of this investigation was to describe baseline VFQ-25 visual function scores, to evaluate whether they differ from previous phase III clinical trials, to determine which contributing factors (e.g. indication, age, gender) affect VFQ-25 scores and to identify its impact on driving.

**Methods:**

The non-interventional OCEAN study (**O**bservation of treatment patterns with Lu**CE**ntis and real life ophthalmic monitoring, including optional OCT in **A**pproved i**N**dications) is the largest ophthalmic study conducted in Germany, to evaluate the real world situation of patients treated with ranibizumab (NCT02194803). The NEI-VFQ-25 questionnaire was conducted at baseline, months 4, 12 and 24. Descriptive statistics was used to analyse the baseline data. ANOVA was performed to evaluate the impact of various contributing factors on composite and selected subscale scores.

**Results:**

Overall, 4844 (84.1 %) of all 5760 OCEAN patients completed the VFQ-25 questionnaire at baseline. Thereof, 3414 treatment-naïve patients were further analysed. Overall, the VFQ subscore general health was most affected by the ocular disease, followed by general vision. No major differences were detected in comparison to corresponding VFQ-25 scores of previous phase III clinical trials, except in DME patients, or with respect to possible contributing factors. A tendency towards a more decreased VFQ-25 composite score was observed for nAMD, for elderly patients ≥75 years of age, for female patients, for patients with low baseline visual acuity (VA; <50 letters) and for those with statutory health insurance. Indication, age, gender, baseline VA (all *p* <0.01) and the interaction of age and indication, as well as baseline VA and indication (*p* <0.01 each) had a significant impact on composite, general vision and distance vision scores (ANOVA). About 10 % of patients gave up driving due to eyesight issues.

**Conclusions:**

The knowledge of a patient’s subjective disease burden is crucial to understanding anxieties and mental anguish. Additionally, the understanding of the impact of various contributing factors on the VFQ-25 scores and the extent to which they can be influenced help to optimize patient care. It demonstrates the need for medical and mental support by all medical staff, to encourage patients’ compliance with a comprehensive anti-VEGF therapy, to increase BCVA and, consecutively, VFQ-25 scores.

**Trial registration:**

NCT02194803

**Electronic supplementary material:**

The online version of this article (doi:10.1186/s12955-016-0536-1) contains supplementary material, which is available to authorized users.

## Background

In the last decades, major improvements have been made in ophthalmology to objectively assess patients’ impairment due to ocular diseases and to quantify the outcome of various interventions [1]. Besides merely determining visual acuity (VA), which was the gold standard in the past, the additional evaluation of patients’ health-related quality of life (QoL) has come to the fore and is broadly implemented nowadays [2, 3]. The National Eye Institute (NEI) Visual Function Questionnaire (VFQ)-25 is a well-established and validated survey to objectively evaluate patients’ disease burden, to determine vision-related QoL [1, 4, 5] and the psychometric properties of diseases that cause vision loss, like glaucoma [6, 7], cataract [8] and Graves’ ophthalmopathy [9], amongst others [10–14]. Furthermore, it allows an objective judgement of the effect of different interventions like intraocular lens (IOL) implantations [8] or intravitreal anti-vascular endothelial growth factor (VEGF) injections [15–17] on QoL. Hence, the NEI VFQ-25 has been used in almost all phase III trials evaluating the efficacy and safety of intravitreally administered ranibizumab in different indications to objectively determine QoL and, thus, the disease burden for each participating patient [15–17]. QoL measurements are now an integral part of health utility analyses and the drug pricing process in certain countries [18].

The ongoing OCEAN study (**O**bservation of treatment patterns with Lu**CE**ntis and real life ophthalmic monitoring, including optional OCT in **A**pproved i**N**dications), at present the largest German observational study in ophthalmology, was initiated to evaluate the treatment patterns with intravitreally injected ranibizumab in real-world clinical care for all approved indications (neovascular age-related macular degeneration [nAMD], diabetic macular edema [DME], macular edema due to retinal vein occlusion [RVO; central RVO [CRVO] or branch RVO [BRVO]] and myopic chorioretinal neovascularization [mCNV]).

A patient’s current QoL and individual burden of disease is of special interest for all medical staff involved, to optimize not only medical treatment but also emotional attention, both of which directly impact QoL [3]. While phase III, controlled clinical trials generally enroll healthier, strictly selected patients [19], the QoL of real-world patients may be influenced more strongly by contributing factors (e.g. indication, age, gender).

The scope of this report was to evaluate the baseline QoL and disease burden of patients scheduled for intraocular ranibizumab treatment due to various neovascular eye disorders in routine clinical care. The baseline VFQ-25 scores from OCEAN were compared with corresponding results of previous phase III clinical trials, to assess which contributing factors instantly affect QoL and to identify QoL’s impact on driving. Identifying and addressing such factors, if possible, will help to increase overall QoL.

## Methods

The OCEAN study is an open-label, prospective, multicentre, non-interventional study (NIS). Its aim is to observe treatment patterns of intravitreal ranibizumab injections for a period of up to 24 months per patient, including an optional retrospective documentation of any prior anti-VEGF treatment. Recruitment ended in December 2014. The NEI-VFQ-25 questionnaire was administered at baseline, and is repeated at months 4, 12 and 24. The questionnaire covers general health, quality of vision (general vision, near vision, distance vision, peripheral vision, colour vision) and vision-related QoL (driving, ocular pain, role limitations, dependency, social function and mental health). Scores range from 0 (maximally compromised) to 100 (not compromised) [4, 5, 20]. For this report, the baseline VFQ-25 scores and subscale scores were evaluated. The NIS OCEAN was conducted in accordance with the German Drug Law and was approved by the respective Ethics Committee prior to study initiation. All procedures adhered to the ethical standards of the responsible committee as well as the declaration of Helsinki. Written informed consent was obtained from all patients following an explicit explanation of the aim of the study. All participating physicians received compensation for the documentation of each patient in accordance with the official scale of physicians’ fees (http://www.bmg.bund.de/glossarbegriffe/g/gebuehrenordnung-fuer-aerzte-und-zahnaerzte.html). All decisions regarding procedures and treatments were made by the physicians and were not influenced; the frequency and scope of all examinations were to reflect routine clinical practice.

The statistical analysis was conducted according to a predefined statistical analysis plan. The descriptive statistics was performed to identify any variables with predictive validity. Results were also displayed according to appropriate strata. The documented data were examined for plausibility before analysis. An analysis of variance (ANOVA) was performed based on iterative backward selection.

## Results

Overall, 4844 (84.1 %) of all 5760 OCEAN patients were included in the VFQ analysis set, thereof 2096 (43.3 %) male and 2732 (56.4 %) female participants. In 16 cases (0.3 %), gender was not documented. Overall mean age (± standard deviation [SD]) was 74.7 ± 10.4 years (median [1^st^/3^rd^ quartile]: 76.1 years [69.7/81.8]). Information on age was missing for 19 patients (0.4 %). Only 434 (9.0 %) patients were covered by a private health insurance, while 4386 (90.5 %) patients had statutory health insurance (missing data for 24 patients [0.5 %]).

With respect to pre-treatment status, 3414 (70.5 %) patients were classified as treatment-naïve (but with variability regarding the time of diagnosis), 741 (15.3 %) as pre-treated (i.e. patients with prior documented treatment with ranibizumab or any other anti-VEGF-treatment) and 689 (14.2 %) as possibly pre-treated (all other patients). Concerning indications, 3118 (64.4 %) participants had been diagnosed with nAMD, 1033 (21.3 %) with DME, 659 (13.6 %) with RVO, thereof 195 (29.6 %)/113 (17.2 %) with BRVO/CRVO (missing sub-diagnosis for 351 patients [53.3 %]) and 34 (0.7 %) with mCNV.

**Fig. 1:**
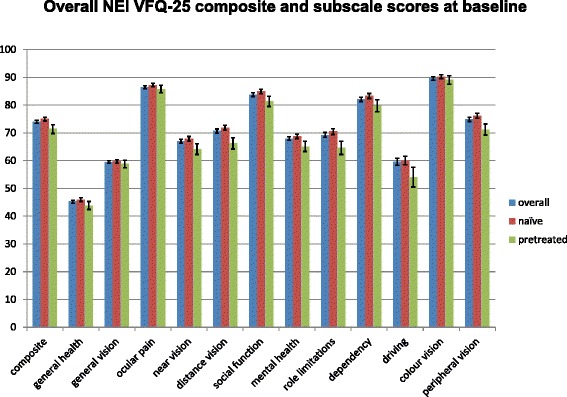
Histogram of vision subscales with mean and confidence interval for all indications evaluated

The overall VFQ-25 composite scores at the OCEAN baseline visit are displayed in Table 1 and Fig. 1. Overall VFQ-25 subscale scores (all indications) are demonstrated in Table 2 and Fig. 1. Overall, general health, general vision and driving seem to be most affected in the OCEAN patients. In contrast, ocular pain and colour vision are least affected, followed by vision-specific social function, vision-specific dependency and peripheral vision. When comparing treatment-naïve and pre-treated patients, the pre-treated patients tend to show more decreased scores for distance vision, vision-specific role limitations and driving.

**Table 1:** Baseline VFQ-25 composite scores of all OCEAN patients as well as stratified data of treatment-naïve and pre-treated patients

	Baseline score [95 % confidence interval]		
VFQ-25	All (*N* = 4844)^a^	Naïve (*N* = 3414)^a^	Pre-treated (*N* = 741)^a^
Composite score	74.0 [73.5; 74.6]	75.0 [74.3; 75.6]	71.4 [69.8; 72.9]

^a^Missing data for <2 % in composite score

**Table 2:** Overall VFQ-25 subscale scores (all indications) as well as stratified data of treatment-naïve and pre-treated patients

	Baseline score [95 % confidence interval]		
VFQ-25 subscale	All (*N* = 4844)^a^	Naïve (*N* = 3414)^a^	Pre-treated (*N* = 741)^a^
General health	45.3 [44.7; 45.8]	45.9 [45.3; 46.6]	43.8 [42.4; 45.3]
General vision	59.5 [59.0; 60.0]	59.7 [59.1; 60.3]	58.8 [57.4; 60.1]
Ocular pain	86.5 [85.9; 87.0]	87.2 [86.6; 87.8]	85.8 [84.4; 87.1]
Near vision	67.0 [66.2; 67.7]	67.8 [67.0; 68.7]	64.1 [62.2; 66.1]
Distance vision	70.7 [69.9; 71.4]	71.9 [71.0; 72.7]	66.2 [64.2; 68.3]
Social function (vision specific)	83.8 [83.1; 84.5]	84.9 [84.1; 85.7]	81.4 [79.5; 83.2]
Mental health (vision specific)	67.9 [67.3; 68.6]	68.8 [68.0; 69.5]	65.1 [63.3; 67.0]
Role limitations (vision specific)	69.2 [68.4; 70.1]	70.5 [69.4; 71.5]	64.6 [62.2; 67.0]
Dependency (vision specific)	82.1 [81.3; 82.9]	83.3 [82.4; 84.2]	79.8 [77.6; 81.9]
Driving	59.6 [58.3; 60.9]	60.0 [58.5; 61.6]	54.1 [50.5; 57.6]
Colour vision	89.6 [89.0; 90.2]	90.3 [89.6; 91.0]	89.1 [87.5; 90.6]
Peripheral vision	74.9 [74.1; 75.6]	76.2 [75.3; 77.1]	71.2 [69.2; 73.2]

^a^Missing data for <3 % in subscale scores; except for driving, which is only reported for those subjects who reported to currently drive or to have given up driving mainly due to eyesight

All further analyses presented in this report focus on the treatment-naïve patients, because the baseline VFQ-25 scores of naïve patients represent a more precisely defined and homogeneous group.

**Fig. 2:**
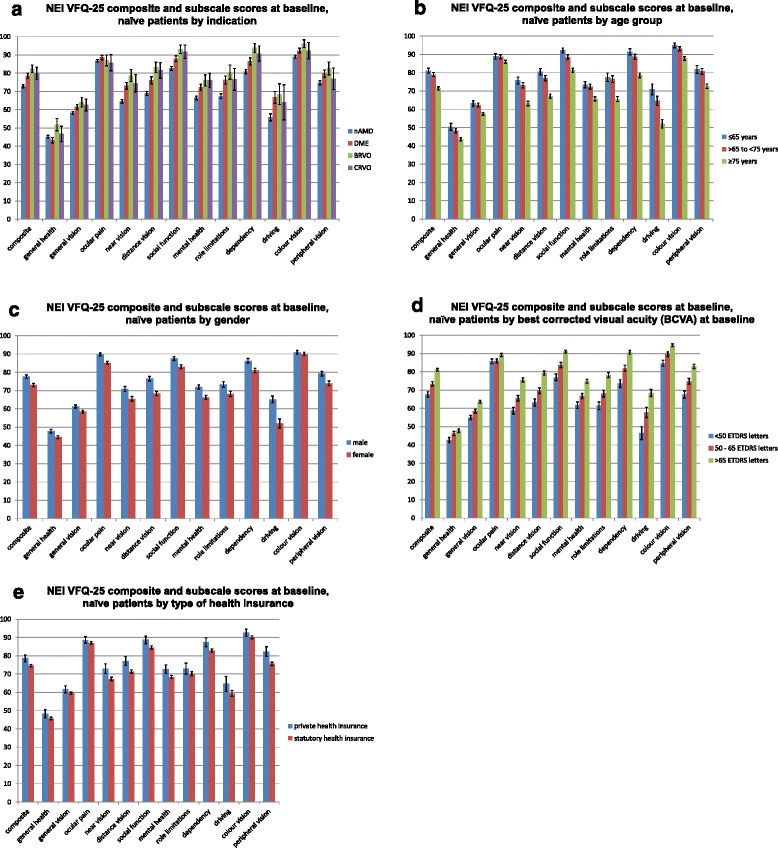
**a** Differences in baseline VFQ-25 scores by indication (nAMD/DME/RVO). **b** Differences in baseline VFQ-25 scores by age. **c** Baseline VFQ-25 scores by gender. **d** Variations in baseline VFQ-25 scores in treatment-naïve patients dependent on baseline BCVA. **e** Baseline VFQ-25 scores of treatment-naïve patients by health insurance type

Figure 2a presents the differences in baseline VFQ-25 scores by indication (nAMD/DME/RVO [BRVO/CRVO]) in treatment-naïve patients. The analysis included 2264 patients (66.3 %) with nAMD, 655 (19.2 %) with DME, 139 (4.1 %) with BRVO, and 80 (2.3 %) with CRVO (missing RVO classification in 253 patients (7.4 %)). The mCNV patients are not analysed in detail here, as this population is too small to allow reliable conclusions. Patients suffering from RVO of any type tend to show the highest VFQ-25 scores, followed by DME and nAMD patients. The most decreased visual function is detected for nAMD patients, with significantly worse results (non-overlapping 95 % confidence intervals) in the composite score and in several subscale scores (general vision, near vision, distance vision, and the vision-specific scores social function, mental health, role limitations and dependency) compared to the other indications. Overall, however, no major differences between the indications can be observed.

The differences in baseline VFQ-25 scores by age are displayed in Fig. 2b. Among all treatment-naïve patients, 528 (15.5 %) are younger than 65 years and, thus, of working age. A total of 943 (27.6 %) participants are between 65 and 75 years of age (early retirement phase) and the majority (1928 [56.5 %] patients) are 75 years and older. There is a tendency towards a decrease in visual function with increasing age. In comparison to both younger age groups, the composite score and all subscale scores are significantly more affected for patients aged 75 years and older (non-overlapping 95 % confidence intervals).

The baseline VFQ-25 scores by gender are shown in Fig. 2c. A total of 1496 (43.8 %) treatment-naïve participants are male and 1904 (55.8 %) are female (missing gender in 14 cases [0.4 %]). No major differences between genders are detected. A tendency towards a more decreased visual function for females is observed, as significantly reflected in the composite score and most subscale scores (non-overlapping 95 % confidence intervals), except for colour vision.

Variations in baseline VFQ-25 scores in treatment-naïve patients dependent on the baseline best-corrected VA (BCVA) of the treated eye at baseline are shown in Fig. 2d. A total of 915 participants (26.8 %) have a BCVA of less than 50 letters, 1065 (31.2 %) of 50 to 65 letters and 1404 (41.1 %) of more than 65 letters. There is a tendency towards a more decreased visual function for patients with lower baseline BCVA, which is significantly reflected in the composite score and most subscale scores except for general health and ocular pain (non-overlapping 95 % confidence intervals).

The baseline VFQ-25 scores of treatment-naïve patients by health insurance type are displayed in Fig. 2e. A total of 323 patients (9.5 %) are covered by private health insurance and 3072 (90.0 %) by statutory health insurance (missing data for 19 [0.6 %] patients). Overall, no major differences are visible between the health insurance types. There is a tendency towards a more decreased visual function for statutorily insured patients, as significantly demonstrated by a decreased composite score and several subscale scores (near vision, distance vision, peripheral vision and the vision-specific scores social function, mental health and dependency (non-overlapping 95 % confidence intervals).

The above comparisons of baseline VFQ-25 scores are based on the single parameters indication, age, gender, baseline BCVA and health insurance type. An additional ANOVA including all these parameters (except health insurance type), as well as their pairwise interactions was performed for the composite score and for the subscale scores general vision and distance vision. Statistical significance could be shown for all single parameters (all *p* <0.01), for the interaction of age and indication (*p* <0.01), as well as for the interaction of baseline BCVA and indication (*p* <0.01). Results are comparable for the three analysed VFQ-25 scores.

**Fig. 3:**
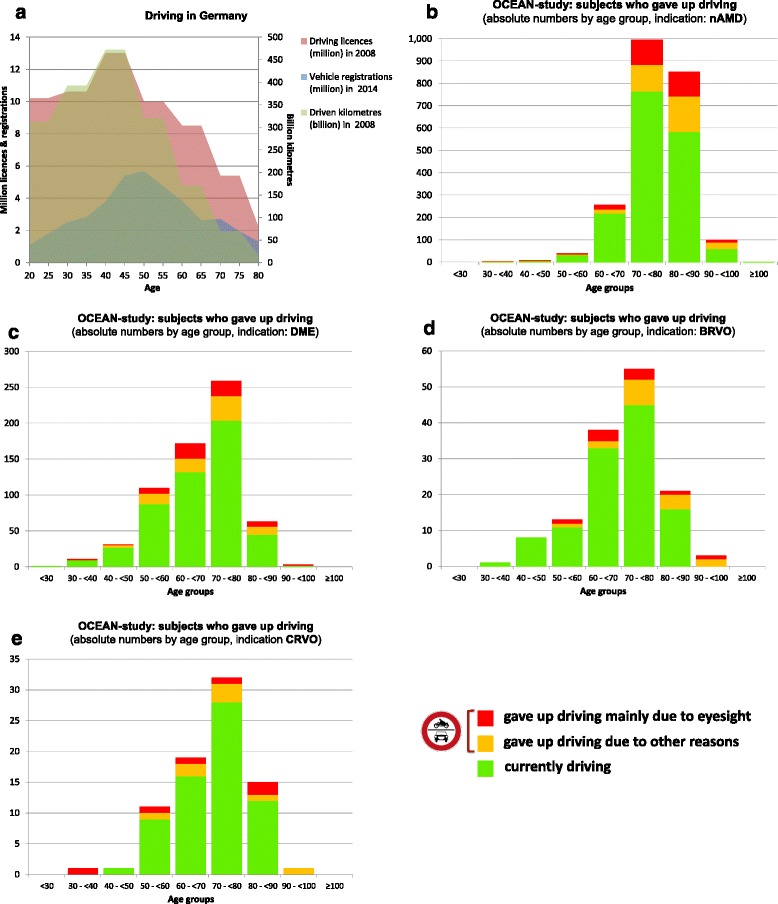
**a** Driving licences, vehicle registrations and driven kilometres in Germany by age. **b–e** Age distribution of OCEAN participants who gave up driving generally or mainly due to eyesight issues, by indication

Figure 3a presents driving licences, vehicle registrations and driven kilometres in Germany by age [21, 22]. Table 3 and Fig. 3b to e show the age distributions of the OCEAN participants who gave up driving generally or mainly due to eyesight issues, by indication. In Germany the maximum of driving licences, vehicle registrations and driven kilometres can be found in the age class between 40 and 45 years, decreasing constantly in the older age classes with a distinct reduction from 65 years on. In the OCEAN study, the number of patients who gave up driving generally increases from between 50 and 60 years of age onwards, reaching a maximum in the age classes between 80 and 100. The leading cause for giving up driving is mainly eyesight. This is generally most pronounced for nAMD patients, followed by DME, BRVO and CRVO.

**Table 3:** Age distribution of OCEAN participants who gave up driving generally or mainly due to eyesight issues, by indication

		Age group								
Indication		<30	30 - <40	40 - <50	50 - <60	60 - <70	70 - <80	80 - <90	90 - <100	≥100
nAMD	n	0	4	7	39	257	996	852	99	1
	Gave up driving… [n (%)]	0 (0 %)	2 (50 %)	1 (14 %)	7 (18 %)	38 (15 %)	232 (23 %)	269 (32 %)	40 (40 %)	0 (0 %)
	…mainly due to eyesight [n (%)]	0 (0 %)	1 (25 %)	1 (14 %)	4 (10 %)	20 (8 %)	112 (11 %)	110 (13 %)	11 (11 %)	0 (0 %)
DME	n	1	11	31	110	172	259	63	3	0
	Gave up driving… [n (%)]	0 (0 %)	2 (18 %)	4 (13 %)	23 (21 %)	40 (23 %)	55 (21 %)	18 (29 %)	1 (33 %)	0 (0 %)
	…mainly due to eyesight [n (%)]	0 (0 %)	2 (18 %)	1 (3 %)	8 (7 %)	21 (12 %)	21 (8 %)	7 (11 %)	1 (33 %)	0 (0 %)
BRVO	n	0	1	8	13	38	55	21	3	0
	Gave up driving… [n (%)]	0 (0 %)	0 (0 %)	0 (0 %)	2 (15 %)	5 (13 %)	10 (18 %)	5 (24 %)	3 (100 %)	0 (0 %)
	…mainly due to eyesight [n (%)]	0 (0 %)	0 (0 %)	0 (0 %)	1 (8 %)	3 (8 %)	3 (5 %)	1 (5 %)	1 (33 %)	0 (0 %)
CRVO	n	0	1	1	11	19	32	15	1	0
	Gave up driving… [n (%)]	0 (0 %)	1 (100 %)	0 (0 %)	2 (18 %)	3 (16 %)	4 (13 %)	3 (20 %)	1 (100 %)	0 (0 %)
	…mainly due to eyesight [n (%)]	0 (0 %)	1 (100 %)	0 (0 %)	1 (9 %)	1 (5 %)	1 (3 %)	2 (13 %)	0 (0 %)	0 (0 %)

Overall, the mean BCVA (± SD) of the treated eye is 58.4 ± 18.3 letters in patients driving at study baseline. The BCVA is lower in patients who gave up driving mainly due to eyesight issues (47.4 letters ± 20.7), who gave up driving mainly due to other reasons (55.1 letters ± 20.0) and who gave up driving due to eyesight and other reasons (48.6 letters ± 23.1).

## Discussion

Being aware of a patient’s subjective QoL and disease burden, as objectively evaluated using the NEI VFQ-25, is crucial to understand a patient’s anxieties, to encourage compliance with initiated intravitreal anti-VEGF therapy and regular monitoring, as well as to support the physician’s and staff’s empathy. Furthermore, knowing the impact of different contributing factors to QoL scores allows the medical staff to estimate whether QoL can be influenced by the medical therapy itself or by supporting emotional attention [3]. For example, if age or gender had a major impact on QoL, these factors would be non-modifiable. On the other hand, if VA was found to be a key factor for QoL scores, there would be a good chance to improve these scores by adequate medical treatment and emotional attention.

The NEI-VFQ-25 measures the influence of visual disability on general health domains such as emotional well-being, social functioning and daily visual function. It allows judgement of the extent to which eye diseases impact anxiety, routine activities and the interaction with family and friends [20]. Previous research demonstrated a correlation between decreased visual function and depression development, which in turn hampers daily activity [23]. This was identified as one main reason for the high incidence of depressive disorders in patients with advanced AMD, comparable to patients with life-threatening diseases such as cancer or cerebrovascular diseases [24, 25]. In the light of these correlations, baseline VFQ-25 scores can help medical staff to provide patients with optimized medical treatment and emotional attention.

In the NIS OCEAN, 84 % of the large patient population performed the VFQ-25 at baseline and, thus, the results likely reflect the true situation in routine clinical care. In general, patients might be afraid of reporting their significantly reduced QoL and major disease burden to the examining and treating medical staff, especially at a higher age [26]. However, this is important information for judging patients’ mental strain, for providing psychological support and for increasing compliance, which will result in better visual outcomes and, consequently, in an improved QoL [23] during and after intraocular anti-VEGF treatment.

Previous reports evaluated the impact of treatment outcomes of better-seeing (BSE) and worse-seeing eyes (WSE) on VFQ-25 results. It was shown that an impaired QoL is even evident if only one eye is affected by the underlying disease, with unimpaired vision of the BSE [27]. Thus, the WSE appears to have a noticeable influence on QoL, contrary to the common assumption that the BSE mostly determines QoL. However, the “AMAs Guides to the Evaluation of Permanent Impairment” weighted average gives a factor of 0.75 to the BSE and of 0.25 to the WSE [26], stressing the importance of the BSE [28]. Impaired vision in the WSE per se might reduce QoL due to fears of losing vision in the BSE as well [27]. QoL can even be affected prior to vision loss due to a patient’s emotional reaction to diagnosis and treatment [29]. A recent study finally demonstrated that improvements in QoL scores were not significantly associated with the treatment of either WSE or BSE. Only VA improvements in the treated eye, irrespective of WSE or BSE status, were associated with an increase in QoL [15, 30]. Unfortunately, there is no distinction between WSE and BSE in the OCEAN study.

The comparison of the OCEAN VFQ-25 baseline scores with the results of former published phase III clinical trials of intravitreal ranibizumab for the treatment of nAMD, DME, and macular edema due to RVO is of clinical interest (Additional file 1: Table S1). Baseline VFQ-25 outcomes in MARINA/ANCHOR disclosed overall scores of 57.6/52.0 for the BSE as well as 78.3/79.4 for the WSE. This in turn emphasizes the impact of VA reduction on QoL in the BSE [15]. The mean OCEAN baseline score (irrespective of the treatment of BSE or WSE) of 72.8 in treatment-naïve nAMD patients is in between these MARINA/ANCHOR scores but closer to the results for the WSE. The same interrelation can be obtained for almost all subscale scores [15]. In patients suffering from DME, the overall VFQ-25 composite score at baseline was about 66 (BSE: 52 to 60) in a recent exploratory post hoc analysis of the RISE and RIDE data [16]. The corresponding baseline score of 78.5 in treatment-naïve DME patients in the OCEAN study is distinctly higher, even if compared to the composite score of 69 to 74 for the WSE in RISE/RIDE [16]. This correlation is seen for almost all subscale scores, especially for social function, mental health and dependency [16]. Patients with macular edema due to RVO (CRVO or BRVO) showed baseline VFQ-25 composite scores of between 76 and 77 in the BRAVO/CRUISE trials [17]. In OCEAN, the analogous scores are slightly higher with 79.9 to 82.5 points. Comparable results are seen for most subscale scores, while dependency, mental health and role limitations have notably higher scores in OCEAN.

In summary, these data show that VFQ-25 composite scores and especially emotional well-being as well as social functioning subscale scores (e.g. social functioning, mental health, role limitations and dependency) have increased from the initial phase III clinical trials of intravitreally applied anti-VEGF substances to today’s routine clinical care. This could on the one hand reflect the awareness and the increased expectations of patients suffering from neovascular eye disorders with respect to this innovative treatment option. This is supported by the comparison of VFQ-25 baseline scores from the OCEAN study with QoL evaluations prior to the era of anti-VEGF therapies. For instance, VFQ-25 scores were tremendously lower in patients with nAMD when only PDT or macular surgery were available [23], in patients with diabetes when focal or grid laser treatment were the only options to treat DME [31], as well as in RVO-affected eyes [32] when merely laser treatment and surgery for severe cases (e.g. vitrectomy in combination with radial optic neurotomy [RON] or arterio-venous sheathotomy [AVS9] [33]) were available. Thus, intravitreally injected anti-VEGF substances have, besides anatomic and functional considerations, a major impact on social and mental aspect and can distinctly increase patient QoL. On the other hand, these increased VFQ-25 composite and subscale scores herein might be attributed to a better baseline BCVA as compared with the pivotal phase III clinical trials (Additional file 2: Table S2).

All clinical trials mentioned above demonstrated not only medical improvements (e.g. in BCVA, central retinal thickness [CRT]), but also a significant increase in QoL scores during and after anti-VEGF treatment [15–17], further supporting the beneficial effect of intravitreally applied anti-VEGF substances. This effect can be useful to reduce anxieties and to encourage patients’ compliance.

As analysed herein, the OCEAN baseline VFQ-25 scores show no major differences between indications overall. However, a tendency towards a more decreased visual function for nAMD in comparison to DME and RVO patients is noted. As the indication per se cannot be modified, there is almost no scope for QoL improvement in this respect. Especially nAMD patients with severe bilateral affection have serious difficulties in performing most vision-dependent daily activities (e.g. reading, seeing well close up and navigating around their homes and neighbourhoods) due to loss of central vision [5]. This causes distinctly low subscale scores for dependency, role limitations, mental health and social function, all of which demonstrate the potential isolating effect of bilateral severe AMD [5]. Considering this, medical staff should encourage patients to demand psychological support, if needed, or to attend meetings of specialized support groups, for instance. Patients suffering from DME showed comparable scores to nAMD patients, but a significantly worse VFQ-25 composite score in comparison to glaucoma or cataract patients and in comparison to a healthy reference group [20]. Furthermore, DME-affected type II diabetics were significantly more affected overall than type I diabetics [20]. The severity of peripheral retinal alterations (diabetic retinopathy, DR) showed an immediate impact on QoL scores. Especially the progression from unilateral to bilateral non-proliferative diabetic retinopathy (NPDR) caused most substantial decreases [34]. If DR progresses to the proliferative variant (PDR), a patient can lose 25 to 30 VFQ-25 points in comparison to NPDR patients, especially in the “mental health” category [26]. Finally, lower VFQ-25 scores were independently associated with poorer VA, older age and a history of loss of tactile sensation as a marker of advanced systemic diabetic alterations [35]. Another publication demonstrated that, in eyes with BRVO, QoL was significantly less affected than in nAMD and DME eyes. This difference was attributed to the one-sided event in RVO; whereas diabetic changes and nAMD most frequently occur on both sides during the course of disease [36].

With respect to the impact of patients’ age on QoL, no major differences are found between the age groups in OCEAN. There is a tendency towards decreased visual function with increasing age, particularly in patients 75 years or older. This observation is supported by previous investigations [1, 5], demonstrating a significant age-dependency of VFQ-25 scores by approximately 1.0 point for every life decade [1]. In patients suffering from severe bilateral AMD, QoL tended to correlate negatively with increasing patient age and duration of vision loss, especially for quality of vision and vision-related QoL subscales [5]. Depression might occur more frequently in older patients and it was formerly demonstrated that depression interferes with VFQ-25 scores, irrespective of VA impairment [37]. This is of importance because the proportion of older patients will increase in the future [38].

In OCEAN, no major differences between VFQ-25 scores by gender are detected. A tendency towards a more decreased visual function for females is observed. However, in the OCEAN study, females are older than males (≥75 years: 748 [50 %] males, 1179 [61.9 %] females at baseline) and this age difference might be reflected in the gender difference. Other investigations reported better VFQ-25 scores in females than in males [39].

With regard to the impact of baseline BCVA on VFQ-25 scores, no major differences between groups are detected in OCEAN. There is a tendency towards a more decreased visual function for patients with a lower baseline VA. This observation is in accordance with former findings describing a major impact of BCVA on QoL [19, 30, 35, 40, 41]. A summary of the mean baseline BCVA values in OCEAN and selected clinical trials is provided in Additional file 2: Table S2. As BCVA at the beginning of any therapy might only be influenced marginally by the medical staff involved, attention should be shifted towards the following intervention period. Former investigations demonstrated that in all neovascular diseases discussed herein, BCVA increased significantly during anti-VEGF treatment [42–46], along with significant improvements in VFQ-25 scores [15–17, 19, 40, 47, 48]. This in turn can nourish patients’ hope that the impaired BCVA (and QoL) will improve further. Treatment outcomes are often less good in routine clinical care than in phase III clinical trials because the treatment population is more diverse and treatment tends to be less intense [19, 30, 40, 49]. Nevertheless, a strict anti-VEGF treatment, possibly including a treat-and-extend strategy [41, 50], has the potential to significantly increase the medical and mental condition of patients, to reduce anxiety, prevent depression development and to support therapeutic compliance. An analysis of the VFQ-25 changes in OCEAN during and after the treatment period, in relation to therapeutic success, will be reported separately after completion of the ongoing study.

Overall, no major differences of VFQ-25 scores are detected in OCEAN with respect to the type of health insurance (private versus statutory health insurance). However, there is a tendency towards a more decreased visual function for statutorily insured patients. This is of interest because the type of health insurance might have an impact on QoL scores due to patients’ expectations to receive better medical care in private health insurance. However, this could not be demonstrated herein and is only addressable to some extent.

The ANOVA analysis shows that, besides age and gender, the eye-specific contributing factors (indication, baseline VA, interactions of age and indication as well as baseline VA and indication) have a significant impact on the composite as well as on general and distance vision subscale scores. Especially the general and distance vision scores, as assessed by an ophthalmologist, are of fundamental importance for patients’ ability to drive.

### VFQ-25 and driving

In Germany the numbers of driving licences, vehicle registrations and driven kilometres decrease steadily with age from 45 years of age on, with a distinctive reduction from 65 years on [21, 22]. This is in line with the results of the baseline evaluation of treatment-naïve subjects who gave up driving as evaluated with the VFQ-25 questionnaire in the ongoing OCEAN study. Here, while 10 % of (treatment-naïve) participants in the age group of 40 to <50 years gave up driving, the percentage increases steadily with age, to 41 % in the 90 to <100 years age group (age groups below 40 and above 100 years are not discussed due to the low numbers of patients). Remarkably, the percentage of patients who attributed their giving up driving mainly to eyesight issues does not increase as much with age. Here, the question arises which other reasons beside eyesight are responsible for giving up driving. Unfortunately, the VFQ-25 questionnaire does not address this issue. Therefore, it seems to be essential that future studies evaluating QoL should focus on this unanswered question. The discrepancy between the overall increasing percentage of subjects who gave up driving and the less increasing percentage of patients relating this mainly to eyesight issues remains unexplained. An increasing percentage of eyesight reasons alongside with an increasing overall percentage would have been expected. By speculation, older patients might be or might feel adjusted to a reduced BCVA and thus do not realize the need to stop driving. This should be a reminder for treating ophthalmologists that they are obliged to inform patients not to drive if they are considered unable to do so safely [51]. This seems to be of particular importance for patients suffering from a potential bilateral neovascular eye disorder. In OCEAN, nAMD and DME show a higher and increasing percentage of patients who gave up driving in general than unilateral diseases like BRVO and CRVO. Finally, the OCEAN patients who continued driving have a considerably better BCVA in the eye involved than those who gave up. Unfortunately, BCVA measurements of the fellow eye and/or binocular BCVA measurements are not available.

## Conclusions

In summary, the analyses of the baseline VFQ-25 scores representing patients’ QoL as reported herein show that general health seems to be affected the most, followed by general vision in all approved indications. Ocular pain and colour vision seem to be least affected by the ocular disease, followed by vision-specific social function and vision-specific dependency. These tendencies can be seen across all subgroup analyses. No major differences between VFQ-25 data by predefined strata (indication, age, gender, baseline VA and type of health insurance) were observed. However, a significantly decreased VFQ-25 composite score was seen for the indication nAMD, for elderly patients ≥75 years of age, for female patients, for patients with low baseline VA (<50 letters) and for those with statutory health insurance. In contrast, significantly better visual function was seen for younger (≤65 years) patients, male patients, and patients with a baseline VA of >65 letters or with private health insurance. The knowledge of a patient’s subjective disease burden is crucial to understand the patient’s anxieties and mental anguish. Additionally, the understanding of the impact of various contributing factors on the VFQ-25 scores and the extent to which they can be influenced will help to understand each individual patient. This demonstrates the need for medical and mental support by all medical staff to encourage patients’ compliance with a comprehensive anti-VEGF therapy, because this in turn will increase BCVA and, consecutively, QoL levels. All medical staff is required to focus on these issues to deliver the best individual patient-centred care before, during and after intravitreal anti-VEGF treatment. This will increase patients’ QoL and reduce any negative consequences of the diagnosis and the treatment burden of intraocular anti-VEGF injections.

## Additional files

Additional file 1: Table S1. Mean baseline NEI VFQ-25 composite scores and study periods for OCEAN (treatment-naïve participants) and for pivotal clinical trials of ranibizumab, by indication (nAMD, DME, BRVO, CRVO). (DOCX 23 kb) Additional file 2: Table S2. Mean baseline visual acuity values in OCEAN (treatment-naïve participants) and in pivotal clinical trials of ranibizumab, by indication (nAMD, DME, BRVO, CRVO). (DOCX 23 kb)

## References

[1] Hirneiss C, Schmid-Tannwald C, Kernt M, Kampik A, Neubauer AS (2010). The NEI VFQ-25 vision-related quality of life and prevalence of eye disease in a working population. Graefes Arch Clin Exp Ophthalmol.

[2] Gall C, Mueller I, Kaufmann C, Franke GH, Sabel BA (2008). [Visual field defects after cerebral lesions from the patient’s perspective: health- and vision-related quality of life assessed by SF-36 and NEI-VFQ]. Nervenarzt.

[3] Hirneiss C, Neubauer AS, Welge-Lüssen U, Eibl K, Kampik A (2003). [Measuring patient’s quality of life in ophthalmology]. Ophthalmologe.

[4] Mangione CM, Lee PP, Gutierrez PR, Spritzer K, Berry S, Hays RD, Investigators NEIVFQFT (2001). Development of the 25-item National Eye Institute Visual Function Questionnaire. Arch Ophthalmol.

[5] Cahill MT, Banks AD, Stinnett SS, Toth CA (2005). Vision-related quality of life in patients with bilateral severe age-related macular degeneration. Ophthalmology.

[6] van Gestel A, Webers CA, Beckers HJ, van Dongen MC, Severens JL, Hendrikse F, Schouten JS (2010). The relationship between visual field loss in glaucoma and health-related quality-of-life. Eye (Lond).

[7] Lisboa R, Chun YS, Zangwill LM, Weinreb RN, Rosen PN, Liebmann JM, Girkin CA, Medeiros FA (2013). Association between rates of binocular visual field loss and vision-related quality of life in patients with glaucoma. JAMA Ophthalmol.

[8] Alió JL, Plaza-Puche AB, Piñero DP, Amparo F, Rodríguez-Prats JL, Ayala MJ (2011). Quality of life evaluation after implantation of 2 multifocal intraocular lens models and a monofocal model. J Cataract Refract Surg.

[9] Bradley EA, Sloan JA, Novotny PJ, Garrity JA, Woog JJ, West SK (2006). Evaluation of the National Eye Institute visual function questionnaire in Graves’ ophthalmopathy. Ophthalmology.

[10] Hall TA, McGwin G, Searcey K, Xie A, Hupp SL, Owsley C, Kline LB (2006). Health-related quality of life and psychosocial characteristics of patients with benign essential blepharospasm. Arch Ophthalmol.

[11] Schweitzer KD, Eneh AA, Hurst J, Bona MD, Rahim KJ, Sharma S (2011). Visual function analysis in acute posterior vitreous detachment. Can J Ophthalmol.

[12] Vitale S, Goodman LA, Reed GF, Smith JA (2004). Comparison of the NEI-VFQ and OSDI questionnaires in patients with Sjögren’s syndrome-related dry eye. Health Qual Life Outcomes.

[13] Clemons TE, Gillies MC, Chew EY, Bird AC, Peto T, Figueroa M, Harrington MW, Group MTR (2008). The National Eye Institute Visual Function Questionnaire in the Macular Telangiectasia (MacTel) Project. Invest Ophthalmol Vis Sci.

[14] Qian Y, Glaser T, Esterberg E, Acharya NR (2012). Depression and visual functioning in patients with ocular inflammatory disease. Am J Ophthalmol.

[15] Bressler NM, Chang TS, Suñer IJ, Fine JT, Dolan CM, Ward J, Ianchulev T, Groups MAR (2010). Vision-related function after ranibizumab treatment by better- or worse-seeing eye: clinical trial results from MARINA and ANCHOR. Ophthalmology.

[16] Bressler NM, Varma R, Suñer IJ, Dolan CM, Ward J, Ehrlich JS, Colman S, Turpcu A, Groups RaRR (2014). Vision-related function after ranibizumab treatment for diabetic macular edema: results from RIDE and RISE. Ophthalmology.

[17] Varma R, Bressler NM, Suñer I, Lee P, Dolan CM, Ward J, Colman S, Rubio RG, Groups BaCS (2012). Improved vision-related function after ranibizumab for macular edema after retinal vein occlusion: results from the BRAVO and CRUISE trials. Ophthalmology.

[18] Brown MM, Brown GC (2013). Update on value-based medicine. Curr Opin Ophthalmol.

[19] Finger RP, Guymer RH, Gillies MC, Keeffe JE (2014). The impact of anti-vascular endothelial growth factor treatment on quality of life in neovascular age-related macular degeneration. Ophthalmology.

[20] Hariprasad SM, Mieler WF, Grassi M, Green JL, Jager RD, Miller L (2008). Vision-related quality of life in patients with diabetic macular oedema. Br J Ophthalmol.

[21] Kraftfahrtbundesamt. Fahrzeugulassungen (FZ) Bestand an Kraftfahrzeugen und Kraftfahrzeuganhängern nach Haltern, Wirtschaftszweigen FZ 23. www.destatis.de. Accessed 14 Jul 2015.

[22] ADAC. Fakten & Argumente Kompakt. Mobilität in Deutschland, ausgewählte Ergebnisse. www.adac.de. Accessed 05 Jan 2016.

[23] Lüke M, Ziemssen F, Völker M, Altpeter E, Beutel J, Besch D, Bartz-Schmidt KU, Gelisken F (2009). Full macular translocation (FMT) versus photodynamic therapy (PDT) with verteporfin in the treatment of neovascular age-related macular degeneration: 2-year results of a prospective, controlled, randomised pilot trial (FMT-PDT). Graefes Arch Clin Exp Ophthalmol.

[24] Casten RJ, Rovner BW (2013). Update on depression and age-related macular degeneration. Curr Opin Ophthalmol.

[25] Brody BL, Gamst AC, Williams RA, Smith AR, Lau PW, Dolnak D, Rapaport MH, Kaplan RM, Brown SI (2001). Depression, visual acuity, comorbidity, and disability associated with age-related macular degeneration. Ophthalmology.

[26] Gabrielian A, Hariprasad SM, Jager RD, Green JL, Mieler WF (2010). The utility of visual function questionnaire in the assessment of the impact of diabetic retinopathy on vision-related quality of life. Eye (Lond).

[27] Hirneiss C (2014). The impact of a better-seeing eye and a worse-seeing eye on vision-related quality of life. Clin Ophthalmol.

[28] Linder M, Chang TS, Scott IU, Hay D, Chambers K, Sibley LM, Weis E (1999). Validity of the visual function index (VF-14) in patients with retinal disease. Arch Ophthalmol.

[29] Sharma S, Oliver-Fernandez A, Liu W, Buchholz P, Walt J (2005). The impact of diabetic retinopathy on health-related quality of life. Curr Opin Ophthalmol.

[30] Finger RP, Finger R, Hoffmann AE, Fenwick EK, Wolf A, Kampik A, Kernt M, Neubauer AS, Hirneiss C (2012). Patients’ preferences in treatment for neovascular age-related macular degeneration in clinical routine. Br J Ophthalmol.

[31] Scanlon PH, Martin ML, Bailey C, Johnson E, Hykin P, Keightley S (2006). Reported symptoms and quality-of-life impacts in patients having laser treatment for sight-threatening diabetic retinopathy. Diabet Med.

[32] Deramo VA, Cox TA, Syed AB, Lee PP, Fekrat S (2003). Vision-related quality of life in people with central retinal vein occlusion using the 25-item National Eye Institute Visual Function Questionnaire. Arch Ophthalmol.

[33] Feltgen N, Agostini H, Hansen L (2007). [Surgical treatments for retinal vein occlusion]. Ophthalmologe.

[34] Mazhar K, Varma R, Choudhury F, McKean-Cowdin R, Shtir CJ, Azen SP, Group LALES (2011). Severity of diabetic retinopathy and health-related quality of life: The Los Angeles Latino Eye Study. Ophthalmology.

[35] Klein R, Moss SE, Klein BE, Gutierrez P, Mangione CM (2001). The NEI-VFQ-25 in people with long-term type 1 diabetes mellitus: the Wisconsin Epidemiologic Study of Diabetic Retinopathy. Arch Ophthalmol.

[36] Awdeh RM, Elsing SH, Deramo VA, Stinnett S, Lee PP, Fekrat S (2010). Vision-related quality of life in persons with unilateral branch retinal vein occlusion using the 25-item National Eye Institute Visual Function Questionnaire. Br J Ophthalmol.

[37] Owsley C, McGwin G (2004). Depression and the 25-item National Eye Institute Visual Function Questionnaire in older adults. Ophthalmology.

[38] Franke GH, Gall C (2008). [Quality of life - methodology and clinical practice aspects with a focus on ocular medicine]. Ophthalmologe.

[39] Chatziralli IP, Sergentanis TN, Peponis VG, Papazisis LE, Moschos MM (2013). Risk factors for poor vision-related quality of life among cataract patients. Evaluation of baseline data. Graefes Arch Clin Exp Ophthalmol.

[40] McKean-Cowdin R, Varma R, Hays RD, Wu J, Choudhury F, Azen SP, Group LALES (2010). Longitudinal changes in visual acuity and health-related quality of life: the Los Angeles Latino Eye study. Ophthalmology.

[41] Rakic JM, Leys A, Brié H, Denhaerynck K, Pacheco C, Vancayzeele S, Hermans C, Macdonald K, Abraham I (2013). Real-world variability in ranibizumab treatment and associated clinical, quality of life, and safety outcomes over 24 months in patients with neovascular age-related macular degeneration: the HELIOS study. Clin Ophthalmol.

[42] Rosenfeld PJ, Brown DM, Heier JS, Boyer DS, Kaiser PK, Chung CY, Kim RY, Group MS (2006). Ranibizumab for neovascular age-related macular degeneration. N Engl J Med.

[43] Brown DM, Kaiser PK, Michels M, Soubrane G, Heier JS, Kim RY, Sy JP, Schneider S, Group AS (2006). Ranibizumab versus verteporfin for neovascular age-related macular degeneration. N Engl J Med.

[44] Nguyen QD, Brown DM, Marcus DM, Boyer DS, Patel S, Feiner L, Gibson A, Sy J, Rundle AC, Hopkins JJ (2012). Ranibizumab for diabetic macular edema: results from 2 phase III randomized trials: RISE and RIDE. Ophthalmology.

[45] Brown DM, Campochiaro PA, Singh RP, Li Z, Gray S, Saroj N, Rundle AC, Rubio RG, Murahashi WY, Investigators C (2010). Ranibizumab for macular edema following central retinal vein occlusion: six-month primary end point results of a phase III study. Ophthalmology.

[46] Campochiaro PA, Heier JS, Feiner L, Gray S, Saroj N, Rundle AC, Murahashi WY, Rubio RG, Investigators B (2010). Ranibizumab for macular edema following branch retinal vein occlusion: six-month primary end point results of a phase III study. Ophthalmology.

[47] Inoue M, Arakawa A, Yamane S, Kadonosono K (2014). Intravitreal injection of ranibizumab using a pro re nata regimen for age-related macular degeneration and vision-related quality of life. Clin Ophthalmol.

[48] Mitchell P, Bandello F, Schmidt-Erfurth U, Lang GE, Massin P, Schlingemann RO, Sutter F, Simader C, Burian G, Gerstner O (2011). The RESTORE study: ranibizumab monotherapy or combined with laser versus laser monotherapy for diabetic macular edema. Ophthalmology.

[49] Ziemssen F, Eter N, Fauser S, Bopp S, Radermacher M, Hasanbasic Z, Holz FG, AURA-Studiengruppe (2015). [Retrospective investigation of anti-VEGF treatment reality and effectiveness in patients with neovascular age-related macular degeneration (AMD) in Germany: treatment reality of ranibizumab for neovascular AMD in Germany]. Ophthalmologe.

[50] Arnold JJ, Campain A, Barthelmes D, Simpson JM, Guymer RH, Hunyor AP, McAllister IL, Essex RW, Morlet N, Gillies MC, Group FRBS (2015). Two-year outcomes of “treat and extend” intravitreal therapy for neovascular age-related macular degeneration. Ophthalmology.

[51] DOG. Empfehlung der Deutschen Ophthalmologischen Gesellschaft und des Berufsverbandes der Augenärzte Deutschlands zur Fahreignungsbegutachtung für den Straßenverkehr - Anleitung für die augenärztliche Untersuchung und Beurteilung der Eignung zum Führen von Kraftfahrzeugen. http://www.dog.org/wp-content/uploads/2009/09/Empfehlungsschrift-2011-Endversion.pdf. Accessed 05 Jan 2016.

