# Supplementary material for: Vision-related quality of life in patients receiving intravitreal ranibizumab injections in routine clinical practice: baseline data from the German OCEAN study
Source: Health Qual Life Outcomes. 2016 Sep 20;14:132. doi: 10.1186/s12955-016-0536-1 (PMC5029004; doi:10.1186/s12955-016-0536-1)
Supplement: Additional file 1: Table S1. — Mean baseline NEI VFQ-25 composite scores and study periods for OCEAN (treatment-naïve participants) and for pivotal clinical trials of ranibizumab, by indication (nAMD, DME, BRVO, CRVO). (DOCX 23 kb) [file 12955_2016_536_MOESM1_ESM.docx]

**Supplemental Table 1:** Mean baseline NEI VFQ-25 composite scores and study periods for OCEAN (treatment-naïve participants) and for pivotal clinical trials of ranibizumab, by indication (nAMD, DME, BRVO, CRVO)

| **Indication** Study | **Treatment group** | **n** | **Study duration** | **VFQ composite score at baseline** (mean ± SD) |
| --- | --- | --- | --- | --- |
| **nAMD** |  |  |  |  |
| OCEAN (treatment-naïve patients) | Ranibizumab 0.5 mg | 2245 | 12/2011 – 12/2016 | 72.8 ± 20.2 |
| MARINA [15] | Overall | BSE: 258 | 03/2003 – 12/2005 | BSE: 57.6 ± 16.6  WSE: 78.3 ± 15.9 |
|  |  | WSE: 388 |  |  |
| ANCHOR [15] | Overall | BSE: 107  WSE: 272 | 05/2003 -09/2004 (end of recruitment) | BSE: 52.0 ± 16.3  WSE: 79.4 ± 16.6 |
| **DME** |  |  |  |  |
| OCEAN (treatment-naïve patients) | Ranibizumab 0.5 mg | 646 | 12/2011 – 12/2016 | 78.5 ± 18.1 |
| RISE [16] | Sham injections | BSE: 16  WSE: 82 | 06/2007 – 11/2010 | BSE: 42.4 ± 17.7  WSE: 69.1 ± 18.8 |
|  | Ranibizumab 0.3 mg | BSE: 20  WSE: 80 |  | BSE: 53.9 ± 21.1  WSE: 74.0 ± 16.7 |
|  | Ranibizumab 0.5 mg | BSE: 16  WSE: 86 |  | BSE: 60.3 ± 19.3  WSE: 68.7 ± 18.1 |
| RIDE [16] | Sham injections | BSE: 21  WSE: 76 | 06/2007 – 01/2011 | BSE: 59.7 ± 20.4  WSE: 70.7 ± 18.3 |
|  | Ranibizumab 0.3 mg | BSE: 35  WSE: 71 |  | BSE: 50.8 ± 20.9  WSE: 69.9 ± 18.2 |
|  | Ranibizumab 0.5 mg | BSE: 22  WSE: 78 |  | BSE: 58.6 ± 17.8  WSE: 68.7 ± 18.9 |
| **BRVO** |  |  |  |  |
| OCEAN (treatment-naïve patients) | Ranibizumab 0.5 mg | 138 | 12/2011 – 12/2016 | 82.5 ± 12.2 |
| BRAVO  [17] | Sham injections | 129 | 07/2007 – 05/2009 | 76.5 ± 16.2 |
|  | Ranibizumab 0.3 mg | 133 |  | 76.0 ± 17.0 |
|  | Ranibizumab 0.5 mg | 130 |  | 76.2 ± 15.6 |
| **CRVO** |  |  |  |  |
| OCEAN | Ranibizumab 0.5 mg | 78 | 12/2011 – 12/2016 | 79.9 ± 14.8 |
| CRUISE [17] | Sham injections | 127 | 07/2007 – 06/2009 | 76.7 ± 17.4 |
|  | Ranibizumab 0.3 mg | 130 |  | 77.1 ± 17.0 |
|  | Ranibizumab 0.5 mg | 128 |  | 76.5 ± 16.4 |
| BRVO: branch retinal vein occlusion; BSE: better-seeing eye; CRVO: central retinal vein occlusion; DME: diabetic macular edema; n: number of patients with VFQ measurement at baseline; nAMD: neovascular age-related macular degeneration; NEI: National Eye Institute; SD: standard deviation; VFQ-25: 25-item Visual Function Questionnaire; WSE: worse-seeing eye. | | | | |
