# Supplementary material for: Vision-related quality of life in patients receiving intravitreal ranibizumab injections in routine clinical practice: baseline data from the German OCEAN study
Source: Health Qual Life Outcomes. 2016 Sep 20;14:132. doi: 10.1186/s12955-016-0536-1 (PMC5029004; doi:10.1186/s12955-016-0536-1)
Supplement: Additional file 2: Table S2. — Mean baseline visual acuity values in OCEAN (treatment-naïve participants) and in pivotal clinical trials of ranibizumab, by indication (nAMD, DME, BRVO, CRVO). (DOCX 23 kb) [file 12955_2016_536_MOESM2_ESM.docx]

**Supplemental Table 2:** Mean baseline visual acuity values in OCEAN (treatment-naïve participants) and in pivotal clinical trials of ranibizumab, by indication (nAMD, DME, BRVO, CRVO)

| **Indication** Study | **Treatment group** | **n** | **Visual acuity at baseline** (ETDRS letters analogue, mean ± SD) |
| --- | --- | --- | --- |
| **nAMD** |  |  |  |
| OCEAN (treatment-naïve patients) ^a^ | Ranibizumab 0.5 mg | 2639 | 52.2 ± 21.3 |
| MARINA [42] ^b^ | Sham injections | 238 | 53.6 ± 14.1 |
|  | Ranibizumab 0.3 mg | 238 | 53.1 ± 12.9 |
|  | Ranibizumab 0.5 mg | 240 | 53.7 ± 12.8 |
| ANCHOR [42] ^b^ | Verteporfin | 143 | 45.5 ± 13.1 |
|  | Ranibizumab 0.3 mg | 140 | 47.0 ± 13.1 |
|  | Ranibizumab 0.5 mg | 139 | 47.1 ± 13.2 |
| **DME** |  |  |  |
| OCEAN (treatment-naïve patients) ^a^ | Ranibizumab 0.5 mg | 772 | 61.4 ± 15.1 |
| RISE [44] ^c^ | Sham injections | 127 | 57.2 ± 11.11 |
|  | Ranibizumab 0.3 mg | 125 | 54.7 ± 12.6 |
|  | Ranibizumab 0.5 mg | 125 | 56.9 ± 11.6 |
| RIDE [44] ^c^ | Sham injections | 130 | 57.3 ± 11.2 |
|  | Ranibizumab 0.3 mg | 125 | 57.5 ± 11.6 |
|  | Ranibizumab 0.5 mg | 127 | 56.9 ± 11.8 |
| **BRVO** |  |  |  |
| OCEAN (treatment-naïve patients) ^a^ | Ranibizumab 0.5 mg | 147 | 54.4 ± 21.9 |
| BRAVO [46] ^d^ | Sham injections | 132 | 54.7 ± 12.2 |
|  | Ranibizumab 0.3 mg | 134 | 56.0 ± 12.1 |
|  | Ranibizumab 0.5 mg | 131 | 53.0 ± 12.5 |
| **CRVO** |  |  |  |
| OCEAN (treatment-naïve patients) ^a^ | Ranibizumab 0.5 mg | 84 | 42.4 ± 26.0 |
| CRUISE [45] ^d^ | Sham injections | 130 | 49.2 ± 14.7 |
|  | Ranibizumab 0.3 mg | 132 | 47.4 ± 14.8 |
|  | Ranibizumab 0.5 mg | 130 | 48.1 ± 14.6 |
| ^a^ OCEAN: Different BCVA measurement methods due to non-interventional nature of study; all measurements converted to ETDRS letter analogues for analysis.  ^b^ MARINA and ANCHOR: VA measured with ETDRS chart at a starting distance of 2m.  ^c^ RISE and RIDE: BCVA as measured with ETDRS chart at a starting distance of 4m.  ^d^ BRAVO and CRUISE: BCVA as measured by the procedure described in the ETDRS.  BCVA: best-corrected visual acuity; BRVO: branch retinal vein occlusion; CRVO: central retinal vein occlusion; DME: diabetic macular edema; ETDRS: Early Treatment Diabetic Retinopathy Study; n: number of patients with VA measurement at baseline; nAMD: neovascular age-related macular degeneration; SD: standard deviation; VA: visual acuity. | | | |
